# Genetic polymorphisms associated with telomere length and risk of developing myeloproliferative neoplasms

**DOI:** 10.1038/s41408-020-00356-5

**Published:** 2020-09-01

**Authors:** Matteo Giaccherini, Angelica Macauda, Nicola Sgherza, Juan Sainz, Federica Gemignani, Josè Manuel Sanchez Maldonado, Manuel Jurado, Francesca Tavano, Grzegorz Mazur, Andrés Jerez, Joanna Góra-Tybor, Aleksandra Gołos, Francisca Hernández Mohedo, Joaquin Martinez Lopez, Judit Várkonyi, Raffaele Spadano, Aleksandra Butrym, Federico Canzian, Daniele Campa

**Affiliations:** 1 Department of Biology, University of Pisa, Pisa, Italy; 2 Genomic Epidemiology Group, German Cancer Research Center (DKFZ), Heidelberg, Germany; 3 Division of Hematology, Casa Sollievo della Sofferenza, San Giovanni Rotondo, Italy; 4 U.O.C. Ematologia con Trapianto, Azienda Ospedaliero-Universitaria Consorzionale, Policlinico di Bari, Bari, Italy; 5 Genomic Oncology Area, GENYO, Centre for Genomics and Oncological Research: Pfizer / University of Granada / Andalusian Regional Government, PTS Granada, Granada, Spain; 6 Monoclonal Gammopathies Unit, University Hospital Virgen de las Nieves, Granada, Spain; 7 Pharmacogenetics Unit, Instituto de Investigación Biosanitaria de Granada (Ibs. Granada), Hospitales Universitarios de Granada/Universidad de Granada, Granada, Spain; 8 Department of Medicine, University of Granada, Granada, Spain; 9 Division of Gastroenterology and Research Laboratory, Fondazione IRCCS Casa Sollievo della Sofferenza, San Giovanni Rotondo, Italy; 10 Department of Internal Medicine, Occupational Diseases, Hypertension and Clinical Oncology, Wroclaw Medical University, Wroclaw, Poland; 11 Hematology and Medical Oncology Department, University Hospital Morales Meseguer-IMIB, CIBERER, Murcia, Spain; 12 Department of Hematology, Medical University of Łódź, Łódź, Poland; 13 Department of Clinical Oncology and Chemotherapy, Magodent Hospital, Warsaw, Poland; 14 Hospital 12 de Octubre, H12O-CNIO Hematological Malignancies Clinical Research Unitc Compluntense University, CIBERONC, Madrid, Spain; 15 Third Department of Internal Medicine, Semmelweis University, Budapest, Hungary; 16 Division of Hematology, Casa Sollievo della Sofferenza, San Giovanni Rotondo, Italy; 17 Department of Cancer Prevention and Therapy, Wroclaw Medical University, Wroclaw, Poland

**Keywords:** Risk factors, Epidemiology

## Abstract

Telomere length measured in leukocyte (LTL) has been found to be associated with the risk of developing several cancer types, including myeloproliferative neoplasms (MPNs). LTL is genetically determined by, at least, 11 SNPs previously shown to influence LTL. Their combination in a score has been used as a genetic instrument to measure LTL and evaluate the causative association between LTL and the risk of several cancer types. We tested, for the first time, the “teloscore” in 480 MPN patients and 909 healthy controls in a European multi-center case–control study. We found an increased risk to develop MPNs with longer genetically determined telomeres (OR = 1.82, 95% CI 1.24–2.68, *P* = 2.21 × 10^−3^, comparing the highest with the lowest quintile of the teloscore distribution). Analyzing the SNPs individually we confirm the association between *TERT*-rs2736100-C allele and increased risk of developing MPNs and we report a novel association of the *OBFC1-*rs9420907-C variant with higher MPN risk (OR_allelic _= 1.43; 95% CI 1.15–1.77; *P* = 1.35 × 10^−3^). Consistently with the results obtained with the teloscore, both risk alleles are also associated with longer LTL. In conclusion, our results suggest that genetically determined longer telomeres could be a risk marker for MPN development.

## Introduction

Myeloproliferative neoplasms (MPNs) are a group of hematopoietic stem-cell disorders, characterized by increased production of differentiated cells of the myeloid lineage^1–3^. The main types of MPNs are chronic myeloid leukemia (CML), characterized by the presence of the Philadelphia chromosome, and the *JAK2/CALR/MPL* mutation-related MPNs group, also called Philadelphia chromosome negative, including polycythemia vera (PV), essential thrombocythemia (ET), and primary myelofibrosis (PMF)^4^. The pooled worldwide crude rate incidence of MPNs is 2.58/100,000 per year^5^. MPNs are an elderly disease with a large increase in risk after the age of 60, and are more common in men than in women^3^. Several epidemiologic factors, such as body mass index (BMI), smoking behavior, alcohol consumption, benzene exposure, and physical activity have been associated with one or more specific subgroups of MPNs^1^.

Moreover, there are several evidences for a causative role of somatic mutations in MPN development, such as the V617F mutation in the *JAK2* gene and mutations in *CALR* and *MPL*^6,7^. In addition, common low penetrance variants have been associated with the disease risk, in particular, seven single nucleotide polymorphisms (SNPs) have been identified through three genome-wide association studies (GWAS)^6,8,9^ and, through case–control studies, additional hits such as the 46/1 *JAK2* haplotype^10^ and several SNPs belonging to genes involved in DNA repair, apoptosis, inflammation, and detoxification^11–16^.

In addition, telomere length measured in leukocyte (LTL) has also been investigated as a marker of susceptibility for MPN. LTL is a good proxy for telomere length in other tissues and is relatively stable even across long periods of time^17,18^. LTL has been associated with risk of developing several neoplasms^18–25^.

Five studies have investigated the possible correlation between LTL and MPN risk and all have found a correlation between short telomeres and increased risk of development of the disease^26–31^. Even though the findings seem conclusive, all these studies may suffer from common pitfalls that have to be considered when analysing LTL in relation to neoplastic diseases. All the studies were retrospective and therefore could be influenced by reverse causation bias (i.e., the disease and/or therapies causing the decrease in LTL) as described in a review on LTL association studies^32^. In addition, molecular technologies such as FISH or qPCR are prone to measurement errors due to heterogeneity of biological sample collection, storage, and manipulation^33^. Finally, the studies had a small average sample size (130 cases and 155 controls) that could lead to an overinterpretation of the results.

Telomere length is, at least partially, genetically determined, and 11 SNPs have been identified, through GWAS, to affect LTL^23,34,35^. To overcome the biases of using the direct measure of LTL, the genetic score combination of these 11 SNPs has been successfully used as proxy of genetically determined LTL to investigate its association with various cancer types^23,36–43^. Noteworthy, the associations between LTL and cancer risk have not consistently shown the same trend if the LTL was measured directly through qPCR or through the genetic score^22,23,36,44^. With these premises, we have tested the “teloscore” in 480 MPN cases and 909 controls in a European multi-center case-control study.

## Materials and methods

### Study population

Biological samples were collected in four European countries (Hungary, Italy, Poland, and Spain) and included 480 cases and 909 controls. MPN cases were diagnosed according to the WHO 2008 criteria, and 149 CML, 173 ET, 36 PMF, and 122 PV have been collected. Controls were selected at the same centers where cases were collected and were frequency matched to the cases for age and sex. More in detail: in Spain, Poland and Italy all controls were healthy blood donors arising from the general population that declared to be free of neoplastic pathologies at the moment of the interview. In Hungary, they were recruited at the internal medicine department and were patients diagnosed with any diseases other than malignancies or immunopathological disorders (like arteriosclerosis, liver cirrhosis, myocardial events, stroke, metabolic diseases, nephroliths or colitis). The characteristics of the study population are summarized in Table 1. At diagnosis, all the information about the patients was collected from medical records, namely age, sex, country of origin, and V617F *JAK2* mutation status. Following the guidelines of the Declaration of Helsinki, written informed consent was obtained from each participant. The study was approved by local Institutional Review Boards, namely by the Bioethics Commission of the University of Medical Sciences, Wroclaw, Poland (KB-746/2017); the Bioethical Commission at the Institute of Hematology and Transfusiology, Warsaw, Poland (17/2018); the Regional and Institutional Committee of Science and Research Ethics of Semmelweis University, Budapest, Hungary (263–1/2015); Comitato Etico presso la Fondazione Casa Sollievo della Sofferenza, San Giovanni Rotondo, Italy (MPN-V1.0_20 Nov 17); Comité de Etica de la Investigaciòn, Servicio Andaluz de Salud, Granada, Spain (4092018), by Comité de Ética de la Investigación con Medicamentos del Hospital Universitario 12 de Octubre, Madrid, Spain (18/092); Comité de Ética de la Investigación con Medicamentos del Hospital General Universitario José María Morales Meseguer, Murcia, Spain (EST: 03/19).

**Table 1:** Summary of study population.

	MPN cases	Controls
Country		
Hungary	43	75
Italy	45	182
Poland	212	132
Spain	180	520
Total	480	909
Sex		
Male	43.54%	49.72%
Female	56.46%	50.28%
Median age (25th–75th percentile)	56.46 (45.0–68.5)	52.68 (46.0–60.0)
Disease subtype		
CML	149	
ET	173	
MF	36	
PV	122	

### SNP selection

We selected 11 SNPs (*ZNF676*-rs412658, *TERT*-rs2736100, *CTC1*-rs3027234, *DHX35*-rs6028466, *PXK*-rs6772228, *NAF1*-rs7675998, *ZNF208*-rs8105767, *OBFC1*-rs9420907, *ACYP2*-rs11125529, *TERC*-rs10936599 and *ZBTB46*-rs755017) to be used for the teloscore computation. Each SNP has been discovered by a GWAS and is associated with LTL^23,34,35,45^. Characteristics of the selected SNPs are summarized in Table 2.

**Table 2:** SNPs associated with telomere length.

SNP	Gene	Chr	Position	EA	OA	EAF	*β* (SE)	Base pairs^a^	Discovery *P*_value_	Discovery study
rs412658	*ZNF676*	19	22,359,440	T	C	0.35	0.086 (0.010)	103.2	1.00 × 10^−8^	Mangino^34^
rs8105767	*ZNF208*	19	22,032,639	G	A	0.28	0.064 (0.011)	76.8	1.11 × 10^−9^	Codd^35^
rs3027234	*CTC1*	17	8,232,774	C	T	0.78	0.103 (0.012)	123.6	2.00 × 10^−8^	Mangino^34^
rs9420907	*OBFC1*	10	103,916,707	C	A	0.13	0.142 (0.014)	170.4	7.00 × 10^−11^	Levy^45^
rs755017	*ZBTB46*	20	62,421,622	G	A	0.12	0.019 (0.013)	22.8	6.71 × 10^−9^	Codd^35^
rs6028466	*DHX35*	20	39,500,359	A	G	0.07	0.058 (0.013)	69.6	2.57 × 10^−8^	Haycock^23^
rs7675998	*NAF1*	4	163,086,668	G	A	0.76	0.048 (0.012)	57.6	4.35 × 10^−16^	Codd^35^
rs10936599	*TERC*	3	169,774,313	C	T	0.76	0.100 (0.011)	120	3.00 × 10^−31^	Codd^35^
rs11125529	*ACYP2*	2	54,248,729	A	C	0.11	0.065 (0.012)	78	8.00 × 10^−10^	Codd^35^
rs6772228	*PXK*	3	58,390,292	T	A	0.96	0.041 (0.014)	49.2	3.91 × 10^−10^	Haycock^23^
rs2736100	*TERT*	5	1,286,401	C	A	0.5	0.085 (0.013)	102	4.38 × 10^−19^	Codd^35^

*EA* effect allele, *OA* other allele, *EAF* effect allele frequency in the Caucasian population, *β (SE)* estimate of telomere length variation for each copy of EA.

^a^Base pairs: estimate in base pairs of telomere length variation for each copy of EA, as described in Codd^35^.

### Sample preparation and genotyping

DNA was extracted from whole blood using QIAampR 96 DNA QIAcubeR HT Kit (Qiagen, Hilden, Germany). Genotyping was performed in 384-well plates, using the TaqMan technology (ThermoFisher Applied Biosystems, Waltham MA, USA) according to the manufacturer’s instructions. Each plate contained cases and controls, duplicated samples (8%) were used to check the quality of the genotyping, and negative controls were also added to each plate. Information about the case/control status of the samples was not available to the person performing the genotyping. PCR plates were read on a ViiA 7 Real-Time PCR System and genotypes were called with the QuantStudio software (ThermoFisher Applied Biosystems, Waltham MA, USA).

### Teloscore computation

The “teloscore” was built weighing the effect of each SNP on LTL, based on the knowledge of the allele associated with LTL increase. The estimate of the per-allele effect on LTL for each SNP (expressed in base pairs) was taken from the literature^23^. The score was computed as follows: to each subject and for each genotype a value equal to 0, 1 or 2 was assigned, based on the number of alleles associated with longer telomeres. Then for each SNP in each subject, we multiplied the number of alleles associated with longer telomeres and the per-allele effect on LTL in base pairs. Finally, we summed up the estimated effect of each genotype for each study subject, obtaining a score for each subject. Only a subset of study subjects had a 100% genotyping call rate (330 cases and 687 controls). Therefore, a scaled teloscore was computed with all the subjects included in the study, by dividing the score by the number of genotypes available for each subject. We have not tested the teloscore in our data (i.e., the association between the SNPs and TL measured with RT-PCR), however, the estimate of the effect of each allele has been computed using thousands of individuals in the original studies and we consider it reliable. We will use the wording “genetically determined” telomere length to identify the telomere length predicted by the score throughout the manuscript. Supplementary Table 1 shows an example of how the teloscore was computed.

### Statistical analysis

The association between each SNP and MPN risk was tested with unconditional logistic regression, computing odds ratios (ORs) and 95% confidence intervals (CI), adjusting for age, sex and country of origin. The analysis was performed with allelic (log-additive) and co-dominant models of inheritance, and, considering the Bonferroni correction for multiple testing, the threshold of statistical significance was *P* = 0.05/(11 SNPs × 2 models) = 0.05/22 = 0.0023.

For the teloscore analysis, cases were distributed in quintiles based on the distribution of values in controls. The analyses were performed with unconditional logistic regression computing ORs and 95% CIs, adjusting for age, sex, and country of origin.

### Bioinformatic tools

Each SNP with statistically significant results in the risk association analysis was examined with bio-informatic tools to evaluate a plausible biologic mechanism to explain the association. First, RegulomeDB (https://www.regulomedb.org/regulome-search/)^46^ was used to identify the regulatory potential of each SNP analyzed and the SNPs in LD with them. Then, SNPs with the most interesting regulatory potential were analyzed with the Genotype-Tissue Expression (GTEx) portal web site (https://www.gtexportal.org) to identify potential associations between the SNPs and gene expression (i.e., if the SNPs were expression quantitative trait loci, eQTL)^47^.

## Results

All the selected SNPs were in Hardy-Weinberg equilibrium in the control population. From a total of 503 cases and 929 controls, we excluded 23 cases and 20 controls with a genotyping call rate lower than 72.7% (i.e. we kept samples with at least 8 called SNPs out of 11), leaving 480 cases and 909 controls to use in the statistical analysis with an average genotyping call rate of 97.0%. The concordance rate with duplicated samples was higher than 99%.

### Association of the “teloscore” with MPN risk

We tested the association between the weighted teloscore and MPN risk, using the average scores computed for all the individuals. The observed trend was an increased risk of MPN with longer genetically determined telomere length, comparing individuals in the highest quintile with those in the lowest quintile (OR = 1.82; 95% CI 1.24–2.68; *P* = 2.21 × 10^−3^) (Table 3).

**Table 3:** association between weighted teloscore and MPN risk.

Type of score	Quintiles	OR	95% CI	*p*_value_
Weighted, subjects with 100% call rate	1	1	–	Ref.
	2	1.04	0.65–1.68	0.857
	3	1.4	0.88–2.23	0.153
	4	1.38	0.87–2.17	0.171
	5	1.75	1.12–2.74	**0.014**
	Continuous^a^	1.15	1.04–1.27	**6.00** × **10**^**−3**^
Weighted scaled, all subjects	1	1	–	Ref.
	2	1.25	0.84–1.87	0.271
	3	1.65	1.11–2.45	**0.012**
	4	1.54	1.04–2.27	**0.031**
	5	1.82	1.24–2.68	**2.21** × **10**^**−3**^
	Continuous^a^	1.15	1.05–1.25	**1.55** × **10**^**−3**^

The *p*-value are bold statistically significant.

^a^The unit for the analysis with the continuous variable was the increment of one quintile.

The same trend was observed in the teloscore analyses in the subset of samples with 100% genotyping call rate (330 cases and 687 controls), comparing individuals in the highest quintile with those in the lowest quintile (OR = 1.75; 95% CI 1.12–2.74; *P* = 0.014) (Table 3).

*TERT*-rs2736100 is well-known in the literature to be associated with MPN risk^48^ and it might have pleiotropic effects. We replicated the teloscore analysis without this polymorphism, using the other 10 SNPs to test if the association discovered was driven by the presence of *TERT*-rs2736100. The result observed was in agreement with the result obtained with 11 SNPs (Supplementary Table 2).

### Association between individual SNPs and MPN risk

We observed a statically significant association (considering the Bonferroni-corrected threshold) between *TERT*-rs2736100-C and increased risk of developing MPNs (OR_Heterozygous _= 1.69; 95% CI 1.27–2.22; *P* = 2.89 × 10^−4^) and a suggestive association between *OBFC1*-rs9420907-C and increased risk of MPNs (OR_Allelic _= 1.43; 95% CI 1.15–1.77; *P* = 1.34 × 10^−3^). The results of the analysis are shown in Table 4.

**Table 4:** association between single SNPs and MM risk.

SNP	Gene	Allele	MAF	Allelic model^a^			Codominant model^b^					
		M/m		OR	95% CI	*P*_Value_	OR_Het_	95% CI	*P*_Value_	OR_Hom_	95% CI	*P*_Value_
rs412658	*ZNF676*	C/T	0.35	1.07	0.89–1.28	0.497	1.16	0.88–1.51	0.290	1.07	0.71–1.59	0.746
rs8105767	*ZNF208*	A/G	0.28	1.09	0.90–1.32	0.380	0.97	0.74–1.25	0.797	1.41	0.90–2.20	0.136
rs3027234	*CTC1*	C/T	0.22	1.01	0.83–1.24	0.906	1.18	0.91–1.52	0.207	0.69	0.38–1.24	0.220
rs9420907	*OBFC1*	A/C	0.13	**1.43**	**1.15–1.77**	**1.35** × **10**^**−3**^	1.32	0.99–1.75	0.053	**2.42**	**1.33–4.38**	**3.62** × **10**^**−3**^
rs755017	*ZBTB46*	A/G	0.12	0.86	0.64–1.14	0.291	0.77	0.55–1.06	0.103	1.73	0.54–5.57	0.356
rs6028466	*DHX35*	G/A	0.07	1.12	0.81–1.53	0.495	1.18	0.83–1.67	0.366	0.81	0.19–3.48	0.776
rs7675998	*NAF1*	G/A	0.24	1.05	0.86–1.28	0.645	1.04	0.81–1.33	0.787	1.13	0.66–1.95	0.653
rs10936599	*TERC*	C/T	0.24	1.1	0.89–1.35	0.380	1.14	0.88–1.47	0.316	1.08	0.61–1.94	0.786
rs11125529	*ACYP2*	C/A	0.11	0.89	0.67–1.16	0.374	0.94	0.70–1.26	0.693	0.45	0.13–1.56	0.209
rs6772228	*PXK*	T/A	0.04	1.64	0.97–2.70	0.063	1.56	0.92–2.63	0.099	–	–	–
rs2736100	*TERT*	A/C	0.5	**1.33**	**1.12–1.59**	**1.49** × **10**^**−3**^	**1.69**	**1.27–2.22**	**2.89** × **10**^**−4**^	**1.67**	**1.18–2.38**	**4.21** × **10**^**−3**^

Results in bold are statistically significant.

*MAF* minor allele frequency, *OR* odds ratio, *95% CI* 95% coefficient interval.

^a^Allelic model: M vs m, common allele vs rare allele.

^b^Codominant model: Mm vs MM, heterozygous vs common homozygous; mm vs MM, rare homozygous vs common homozygous.

^c^Dominant model: mm vs Mm + MM, rare homozygous vs heterozygous + common homozygous.

^d^Recessive model: mm + Mm vs MM, rare homozygous + heterozygous vs common homozygous.

### Stratified analyses

Stratified analysis according to *JAK2* mutation status was also performed for the teloscore and for each individual SNP. The results of the teloscore were in agreement with the analysis done regardless of *JAK2* status, with genetically determined longer LTL associated with increased risk of developing MPNs, although the association reached statistical significance only in the *JAK2*+ stratum (Supplementary Table 3). The analysis of the individual SNPs showed two additional borderline associations for *ZNF208*-rs8105767 (*p* = 0.027) and for *ZBTB46*-rs755017 (*p* = 0.037) in the *JAK2*+ stratum and no additional hits (Supplementary Table 4).

### Possible functional effect

We tested with bioinformatic tools the possible functional effect of the individual SNPs associated with risk of MPNs, and we found that RegulomeDB assigns to *OBFC1*-rs9420907 a rank of 3a that indicates that the region where the SNP lies is probably a transcription factor binding site in a DNase sensible region.

## Discussion

Telomeres are structures with a continuous decrease of length in each cell replication, due to the inability to replicate the ends of the chromosome^49^. LTL has been extensively investigated in relation to cancer risk, with several studies suggesting an association between short LTL, as well as long LTL with increased risk and some studies suggesting a null association or a bimodal association^18–25,50^.

Five relatively small retrospective studies have been attempted to identify the relationship between the LTL and MPNs risk through FISH or qPCR. We used a different strategy and computed a genetic score as proxy for LTL and observed that genetically determined longer telomeres were significantly associated with an increased risk of developing MPNs. These results are in contrast with what was observed before i.e. increased risk of MPNs and short LTL. The discrepancies in the findings could be explained by the fact that the direct measure of LTL is plagued by several possible biases. For example, retrospective studies are often influenced by reverse causation bias, especially if chemotherapy is not taken in consideration when estimating the association. In addition, exposure to several risk factors, such as smoking and BMI, is associated with short LTL and tends to confound the association if not accounted for properly. Finally, the decrease of TL is highly correlated with the increase of age, and the measurement of LTL could be affected by bias due to the different age at blood drawing of the cases and controls included in the studies^32,33^. On the contrary, the genetic risk score has the advantage of being independent from study design and from the environmental factors known to be associated with a variation of LTL. The use of genetic proxies to understand the causative correlation between environmental risk factors and outcome has become more and more popular in the last years and it has been instrumental in identifying several of such associations^23,36–43^. The sample size of our study is almost four times larger than the average size of the previous studies (480 vs 130) if considering only the cases and almost five times larger if considering cases and controls combined (1389 vs 285).

We have also analyzed the effect of each SNP and we replicated the well-known association between the *TERT*-rs2736100-C allele with an increased risk of developing MPNs^6,51–53^. The *TERT*-rs2736100-C allele has been consistently associated with longer LTL^21,35^, possibly through the regulation of the telomerase activity^48^. Another potentially interesting association was observed between the *OBFC1*-rs9420907-C allele and increased risk of MPN. The gene product of *OBFC1* is a member of the CST complex, that is involved in telomere maintenance, and it is a member of the alpha accessory factor (AAF), that is involved in the initiation process of DNA replication^54^. It is interesting to note that also the *OBFC1*-rs9420907-C allele is associated with long LTL^45^. Regulome DB assigns to the SNP a rank of 3a indicating a mild possibility of this SNP to be involved in the regulation of nearby genes, however, without in vitro studies, it is not possible, with this level of evidence, to draw a definitive conclusion on the variant. The association of these two SNPs is consistent with what we observed for the score, i.e. the alleles associated with increased MPN risk are also associated with longer LTL. Moreover, an additional SNP *PXK-*rs6772228-T showed the same trend observed for *TERT*-rs2736100-C and *OBFC1*-rs9420904-C, with the T allele, known to be associated with longer LTL, correlated with a non-statistically significant increase of risk of developing MPNs.

A possible limitation of this study is that the controls we selected did not undergo screening to detect myeloid-specific clonal mutations and therefore they might present clonal hematopoiesis, which is a known precursor of hematopoietic malignancies^8,55,56^, however, even if some controls do have clonal hematopoiesis, this could only dilute the true associations between the exposure and the outcome, and not create spurious ones.

Telomere length measured through teloscore could be a possible marker of cells that have the innate tendency to have higher dividing potential. Longer TL may facilitate the acquisition and accumulation of new harmful potential mutations that could increase the risk of developing cancer disease^23^.

Taken together, all these evidences point towards an association between longer LTL and increased risk of developing MPNs despite what has been reported previously.

## Supplementary information

example of teloscore computation

association between weighted teloscore without TERT-re2736100 and MPN risk

teloscore analysis stratified by JAK2 status

individual SNP analyses stratified by JAK2 status

## Data Availability

The primary data for this work will be made available to researchers who submit a reasonable request to the corresponding author, conditional to approval by the centers participating in this study. Data will be stripped from all information allowing identification of study participants.
